# Surgical Management of Traumatic Retinal Detachment with Primary Vitrectomy in Adult Patients

**DOI:** 10.1155/2017/5084319

**Published:** 2017-01-09

**Authors:** Katarzyna Nowomiejska, Tomasz Choragiewicz, Dorota Borowicz, Agnieszka Brzozowska, Joanna Moneta-Wielgos, Ryszard Maciejewski, Anselm G. Jünemann, Robert Rejdak

**Affiliations:** ^1^Department of General Ophthalmology, Medical University of Lublin, Lublin, Poland; ^2^Department of Didactics and Medical Simulation, Human Anatomy Chair, Medical University of Lublin, Lublin, Poland; ^3^Department of Mathematics and Medical Biostatistics, Medical University of Lublin, Lublin, Poland; ^4^Human Anatomy Department, Medical University of Lublin, Lublin, Poland; ^5^Department of Ophthalmology, University of Rostock, Rostock, Germany; ^6^Department of Experimental Pharmacology, Medical Research Centre, Polish Academy of Sciences, Warsaw, Poland

## Abstract

*Purpose.* To evaluate functional and anatomical results of pars plana vitrectomy (PPV) in the retinal detachment (RD) followed by severe eye trauma.* Methods.* Retrospective analysis of medical records of forty-one consecutive patients treated with 23-gauge PPV due to traumatic RD. Age, gender, timing of PPV, visual acuity, and presence of intraocular foreign body (IOFB) and proliferative vitreoretinopathy (PVR) were included in the analysis.* Results.* Mean age of patients was 47 years; the majority of patients were men (88%). Closed globe injury was present in 21 eyes and open globe injury in 20 eyes (IOFB in 13 eyes, penetration injury in 4 eyes, and eye rupture in 3 eyes). Mean follow-up period was 14 months; mean timing of PPV was 67 days. Twenty-seven (66%) eyes had a functional success; 32 eyes (78%) had anatomical success. As a tamponade silicone oil was used in 33 cases and SF6 gas in 8 cases.* Conclusions.* Severe eye injuries are potentially devastating for vision, but vitreoretinal surgery can improve anatomical and functional outcomes. Among analysed pre- and intra- and postoperative factors, absence of PVR, postoperative retinal attachment, and silicone oil as a tamponade were related to significantly improved visual acuity.

## 1. Introduction

Ocular trauma is a major cause of monocular blindness and visual impairment throughout the world. There are data suggesting that at least half a million people are monocularly blind from ocular trauma worldwide [1]. Proper diagnosis and treatment of eye trauma are essential to obtain good anatomical and functional results.

Traumatic retinal detachment (RD) accounts for 10–40% of all detachments [2]. It is supposed that the rate of traumatic RD is at the level of 0.2/10.000 [3]. There is no generally accepted definition of traumatic RD; thus the diagnosis is based on the particular history [4]. RD is seen in up to 30% among all serious eye injuries and has a poor prognosis for successful outcome [5]. RD may accompany both open and close globe injuries [6, 7] but is more prevalent after closed globe injury (about 70–85%) [8]. RD has been reported to occur in up to 30% of open globe injuries and 6–36% of those with posterior segment IOFBs [9].

Pars plana vitrectomy (PPV) has increased the recovery rate in traumatized eyes which previously were deemed inoperable and frequently were enucleated [7]. PPV allows the reconstruction of the posterior segment, controls the healing response [10], and prevents phthisis [11].

The aim of this study was to report functional and anatomical results in patients with trauma related RD treated with PPV in a group of adult patients after open and close globe injuries.

## 2. Materials and Methods

The medical records of forty-one patients having surgery due to traumatic RD during the period from January 2013 to June 2016 in the Department of General Ophthalmology of the Medical University in Lublin, Poland, were retrospectively reviewed. This study followed the tenets of the Declaration of Helsinki. The treatment chosen in the study was a part of a standard care. The study was approved by the local Ethic Committee at the University in Lublin, Poland. The patients gave their written informed consent to participate in the study and publish the data.

The following data were taken into consideration: age, gender, the eye involved, ocular history, type of injury, and ocular findings like initial visual acuity (VA), presence of intraocular foreign body (IOFB), posttraumatic endophthalmitis, choroidal detachment, lens injury, vitreous hemorrhage, follow-up time, and final VA.

The results of imaging studies (B-scan USG, CT) were also obtained in each case. Only eyes with a minimum follow-up time of twelve months were included in this study.

Ocular trauma was classified according to the Ocular Trauma Classification [12, 13]. An open globe injury is defined as a full-thickness defect of the cornea and/or sclera, and open globes are divided into ruptures or lacerations depending on the mechanism of injury (ruptures are caused by blunt objects and lacerations are caused by sharp ones). Lacerations are further subdivided into penetrating injury, intraocular foreign body (IOFB) injury, and perforating injury. A penetrating injury has an entrance wound, an IOFB injury has an entrance wound and a retained IOFB, and a perforating injury has an entrance and an exit wound.

BCVA was assessed with a Snellen chart. BCVA was converted to the logarithm of the minimum angle of resolution (logMAR) units for statistical analysis. In our study, functional success was defined as BCVA equal to or greater than 5/200 [7]; anatomical success was defined as total retinal reattachment at the last follow-up visit.

Statistical computations were performed using STATISTICA 10.0 software (StatSoft, Poland). Qualitative variables were described by percentages and quantitative ones by median ± standard deviation (SD). Wilcoxon test was used for assessment of BCVA results. Differences between groups were assessed using Mann–Whitney *U*-test. Statistical significance was set at 0.05.

## 3. Surgical Procedure

Surgeries were performed in local or general anesthesia. Cataract surgery and iris reconstruction when needed were performed in eyes after closed globe injury, suturing of the corneal, or scleral laceration in case of open globe trauma. Complete 23 G PPV with assistance of triamcinolone and sclera l indentation was performed using constellation system (Alcon, Fort Worth, Texas, US). Indocyanine green was used to dye internal limiting membrane in the posterior pole. Perfluorocarbon liquids were used to attach the retina. Subretinal fluid was drained using extrusion cannula during fluid-air exchange. Laser or cryopexy around the retinal break(s) was done. 5000 cst silicone oil or 20% sulfur hexafluoride (SF6) gas or air was used as a tamponade. Sclerotomies were sutured when needed with vicryl 8.0 sutures.

## 4. Results

There were 36 men (88%) and 5 female (12%) subjects included. The mean age of patients was 49 years (range 25–80 years). Right eye was managed surgically in 56% of patients and the left eye in the remaining 43% of patients. The mean follow-up time was 14 months (range 12–16 months); mean timing of PPV was 67 days (range 1–180 days). Time period from injury to PPV was 22 days in open globe injuries and 102 days in close globe injuries.

Closed globe injury was reported in 21 eyes (51%) and open globe injury in 49% including IOFB in 13 eyes (32%), penetration injury in 4 eyes (10%), and eye rupture in 3 eyes (7%). Vitreous haemorrhage was present in 7 eyes and suprachoroidal haemorrhage in two eyes. PVR was reported in 10% of patients (Table 1). There were 9 total RDs, 8 eyes with giant retinal tears, and 10 eyes with multiple breaks.

Silicone oil was used as a tamponade in 33 eyes (80%) and SF6 gas in 8 eyes (20%) (Table 2). Indications for silicone oil were as follows: presence of PVR, RD with suprachoroidal haemorrhage, multiple breaks, giant retinal tear, and total RD. Thirty-two (78%) of 41 eyes had anatomical success and 27 (66%) functional success. Recurrent RD was observed in 9 eyes (22%). Secondary glaucoma was observed in 14 cases and was managed with antiglaucomatous drops; hypotony was found in one case.

Overall, mean BCVA improved from 1.4 logMAR to 1.25 logMAR (*p* = 0.07). Taking into account particular groups, additional analysis showed that BCVS was significantly (*p* = 0.03) better in eyes without PVR (1.2 logMAR) than in eyes with PVR (1.7 logMAR). Visual acuity was significantly better (*p* = 0.05) after PPV in patients with retina attached postoperatively (from 1.5 to 1.3 logMAR). Visual acuity was significantly improved in eyes with IOFB (1.0 logMAR), rather than in eyes after rupture, closed globe injury, and penetration (1.4 logMAR). BCVA was also significantly improved in eyes with silicone oil as a tamponade (*p* = 0.03).

## 5. Discussion

In the present study we have collected the clinical data of patients treated with 23 G PPV after RD followed by both open and close eye globe injuries. There were many pre-, intra-, and postoperative variables involved; we have found that statistically significant factors were as follows: preoperative PVR, postoperative retinal attachment, and silicone oil as a tamponade.

Comparison of the results in traumatic RD is extremely difficult as the number of variable parameters is large, including extent and localisation of injury and time from injury. Thus, there is a large diversity of clinical pictures resulting from this [14].

Experimental studies have shown that penetrating ocular injuries of posterior segment initiate a sequence of events which can ultimately lead to tractional and/or rhegmatogenous retinal detachment, breakdown of the blood-retinal barrier, initiation of an inflammatory response, and posterior vitreous separation [15, 16]. Proliferative alterations in the region of the vitreous base lead to the annular contraction of the retina; it can be stimulated by the vitreous haemorrhage and incarceration of the vitreous or inadequate wound care. This process can be interrupted by vitrectomy carried out in time.

Studies performed on animal models of ocular blunt trauma indicated that the initiating factor in the pathogenesis of RD is the anteroposterior compression of the globe during impact that leads to compensatory lengthening of the horizontal diameter [17].

Ocular contusion may result in numerous types of retinal breaks, including horseshoe tears, operculated holes, giant retinal tears, macular holes, and retinal dialyses [8, 18]. Sometimes it is difficult to identify the specific cause during clinical examination.

Mechanisms responsible for retinal break formation in closed globe injury may be as follows: vitreous base avulsion, abnormal sites of vitreoretinal adhesion (e.g., lattice degeneration), coup injury, contrecoup injury at a location opposite to the site of impact, or sudden posterior vitreous detachment induction [15]. The sudden acceleration of the vitreous body in closed globe injury may lead to extensive tearing of the retina around the base of the vitreous far out in the periphery.

Surgical repair is indicated as soon as the patient's circumstances allow it. The choice of surgery mainly depends on location of breaks, amount of PVR, and the surgeon's preference.

We suggest performing PPV as it enables removing posttraumatic coaxial opacities as cataract and vitreous haemorrhage in order to obtain a better view into retina with special attention to IOFB and retinal tears; moreover vitreoretinal proliferations and fibrovascular membranes may be also removed with forceps [14]. The presence or absence of choroidal hemorrhage must be determined before surgery by ultrasonography. The infusion cannula (preferably longer 6 mm) should be put with care. Silicone oil injection is preferable if there is coexisting PVR or giant retinal tear, total RD, suprachoroidal haemorrhage, and multiple breaks [19]. Traumatic RD is often complicated with PVR, especially in case of subretinal or vitreous hemorrhage, giant retinal tear, or large wound, when RPE and fibroglial cells have access to vitreous cavity. In our study preoperative PVR was reported in 10% of patients, but silicone oil was used in 80% of patients due to giant retinal tear, total RD, RD with suprachoroidal haemorrhage, and multiple breaks.

Scleral buckling alone may be sufficient after closed globe injury; however there are indications for primary PPV in traumatic RD-vitreous hemorrhage, dislocated lens, PVR, giant retinal tear with everted flap, posterior tear, and subretinal hemorrhage [20].

Due to the complexity of most cases, PPV with tamponade agents as the primary surgical technique is preferred [21]. Almost 40% of the cases will need relaxing retinotomies to reattach the retina [21].

In our study most of the patients were males (88%). Young male individuals are more vulnerable to ocular trauma (80%) probably because of their physical outdoor activities.

In our study mean time from trauma to PPV was 67 days, but it included both open and close globe trauma. It was 22 days in open globe injuries and 102 days in close globe injuries. Relatively long period after closed globe injuries may be explained by the fact that the vitreous body in young individuals is well-formed and posterior hyaloid not detached from the retina. Thus, vitreous limits the progression of RD especially when it is caused by inferior breaks or dialysis. This may be explanation why RSs are not recognized for months after trauma.

Timing of PPV in ocular trauma is a matter of discussion. Ryan and Allen [7] stated that four to ten days after injury seems to be the optimal time for vitrectomy to avoid the hazards of immediate intervention, while removing damaged tissue before serious sequelae occur.

Kuhn presented four options of timing of PPV after severe ocular trauma: early (days 2–4); delayed (days 5–7); late (days 8–14); and very late (past 2 weeks). He concluded that the earlier the vitrectomy, the higher the risk of intraoperative complications, bleeding, wound leakage, and bad visualization. Conversely, the later the vitrectomy, the higher the incidence and severity of postoperative complications, mainly PVR [22, 23]. However, other authors investigated longer time [24] (mean 49 days) and they concluded that a delayed approach is compatible with good visual prognosis in relatively young patients.

The current study is limited by its retrospective methodology and the variability in the included pathology. Trauma cases are inherently unique and thus require an individualized approach to surgical treatment.

## Figures and Tables

**Table 1:** Preoperative data of patients (type of trauma, presence of haemorrhage, and proliferative vitreoretinopathy (PVR), and intraocular foreign body (IOFB)).

	*n*	%
No haemorrhage	32	78,05
Vitreous haemorrhage	7	17,07
Suprachoroidal haemorrhage	2	4,88

Closed globe trauma	21	51,22
IOFB	13	31,71
Open globe trauma	4	9,76
Rupture	3	7,31

No PVR	37	90,24
PVR	4	9,76

**Table 2:** Intraoperative findings during vitrectomy (type of tamponade silicone oil or sulfur hexafluoride (SF6) gas and status of the lens).

	*n*	%
Silicone oil	33	80,49
SF6 gas	8	19,51

Lensectomy	8	19,51
Phacoemulsification	20	48,78
Scleral fixation	1	2,44
Sundmacher lens	1	2,44
Pseudophakia	11	26,83
